# Keyhole versus Sugarbaker techniques in parastomal hernia repair following ileal conduit urinary diversion: a retrospective nationwide cohort study

**DOI:** 10.1186/s12893-021-01228-w

**Published:** 2021-05-03

**Authors:** Elisa Mäkäräinen-Uhlbäck, Jaana Vironen, Markku Vaarala, Pia Nordström, Anu Välikoski, Jyrki Kössi, Ville Falenius, Aristotelis Kechagias, Anne Mattila, Pasi Ohtonen, Tom Scheinin, Tero Rautio

**Affiliations:** 1 Division of Surgery, Medical Research Center, Oulu University Hospital, University of Oulu, Oulu, Finland; 2 Abdominal Center, Helsinki University Hospital, Helsinki, Finland; 3 Division of Surgery, Gastroenterology, and Oncology, Tampere University Hospital, Tampere, Finland; 4 Division of Surgery, Päijät-Häme Central Hospital, Lahti, Finland; 5 Division of Surgery, University of Turku, Turku, Finland; 6 Division of Surgery, Kanta-Häme Central Hospital, Hämeenlinna, Finland; 7 Division of Surgery, Central Finland Central Hospital, Jyväskylä, Finland; 8 Division of Operative Care, Oulu University Hospital, Oulu, Finland; 9 The Research Unit of Surgery, Anesthesia and Intensive Care, University of Oulu, Oulu, Finland

**Keywords:** Cystectomy, Hernia repair, Ileal conduit urinary diversion, Parastomal hernia

## Abstract

**Background:**

Previous research on parastomal hernia repair following ileal conduit urinary diversion is limited. This nationwide cohort study aims to present the results of keyhole and Sugarbaker techniques in parastomal hernia repair in the setting of ileal conduit urinary diversion.

**Method:**

All patients in this cohort underwent primary elective parastomal hernia repair following ileal conduit urinary diversion in four university hospitals and one central hospital in Finland in 2007–2017. Retrospective clinical data were collected from patient registries to compare keyhole and Sugarbaker parastomal hernia repair techniques. The primary outcome was parastomal hernia recurrence during the follow-up from primary surgery to the last confirmed follow-up date of the patient. The secondary outcomes were reoperations during the follow-up and complication rate at 30 days’ follow-up.

**Results:**

The results of 28 hernioplasties were evaluated. The overall parastomal hernia recurrence rate was 18%, the re-operation rate was 14%, and the complication rate was 14% during the median follow-up time of 30 (21–64) months. Recurrence rates were 22% (4/18) after keyhole repair and 10% (1/10) after Sugarbaker repair. Re-operation rates referred to keyhole repair were 22% and Sugarbaker repair 0% during follow-up. The majority of reoperations were indicated by recurrence. Complication rates were 17% after keyhole and 10% after Sugarbaker repair during the 30 days’ follow-up.

**Conclusion:**

The results of parastomal hernia repair in the setting of ileal conduits are below optimal in this nationwide cohort comparing keyhole to Sugarbaker repair in elective parastomal hernia repair. Nonetheless, the Sugarbaker technique should be further studied to confirm the encouraging results of this cohort in terms of recurrence.

## Background

The incidence rate of cystectomies in Finland is 2.7/100,000, leading to 150 new ileal conduit urinary diversions each year [1]. The results of ileal conduit parastomal hernia (PSH) repair are unsatisfactory, with an overall recurrence rate of up to 28% in clinical examination [2]. Small case series that have been published previously include descriptions of stoma relocation [3], a local repair with a mesh [4, 5] and an intra-abdominal mesh repair [6, 7]. Due to a lack of research, there is no recommendation of optimal PSH repair regarding ileal conduits [2, 8].

In the keyhole technique, the parastomal hernia is repaired with a flat mesh with a central hole through which the bowel is brought. In the Sugarbaker technique, the bowel is lateralized and covered with an intra-abdominal mesh [9]. Previous reports of the keyhole and Sugarbaker techniques regarding ileal conduit parastomal hernias are few [6, 7].

As previous research on PSH repair following ileal conduit urinary diversion is scarce, this retrospective cohort study aims to report on the nationwide results of PSH repair by comparing the keyhole and Sugarbaker techniques.

## Methods

Data of all parastomal hernia repairs between January 1st 2007 and December 31st 2017 in five universities and four central hospitals were retrospectively retrieved using International Statistical Classification of Diseases and Related Health Problems (ICD-10) and operation codes. Four university hospitals and two central hospitals had performed elective PSH repairs following ileal conduit urinary diversion during the study period. Only repairs by keyhole and Sugarbaker techniques were included for comparison. One central hospital had not performed any keyhole or Sugarbaker repairs. Additionally, emergency operations and re-operations were excluded. Data collected on specifically designed electronic case report forms included age, body mass index (BMI), indication and date for index ostomy formation, other hernias detected at PSH repair, techniques of the PSH surgery, mesh details, complications, length of stay in the hospital, re-operations and recurrence. The study was approved by the audit departments of all attending hospitals. Parastomal hernia as the primary outcome was defined as one diagnosed by a physician or by the imaging of any modality. The follow-up time is defined from the primary repair to the last confirmed follow-up date in patient records.

### Statistical analysis

The statistics are presented as the mean with the standard deviation (SD) or as the median with the 25th–75th percentiles. As the groups were heterogenous with follow-up time, no statistical analyses were conducted.

## Results

**Fig. 1:**
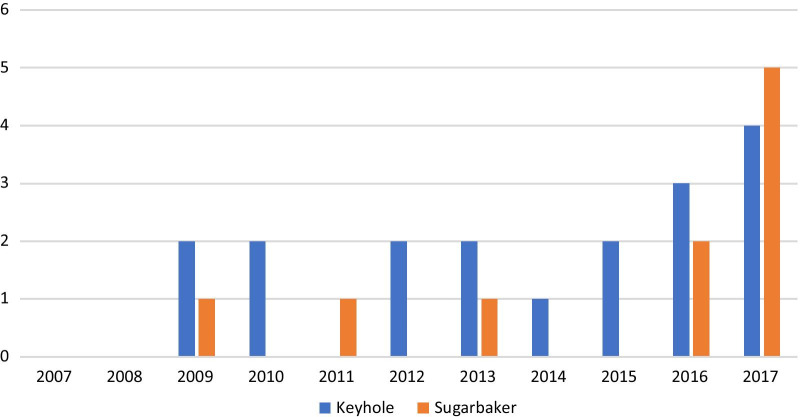
Parastomal hernias repaired following ileal conduit urinary diversion

A cohort of 47 primary PSHs following ileal conduit urinary diversion were operated for primary PSH following ileal conduit urinary diversion in four university hospitals and two central hospitals in Finland in 2007–2017 (Fig. 1). Keyhole repair was performed on 18 patients and Sugarbaker repair on 10 patients in four university hospitals and one central hospital. Additionally, there were seven (15%) onlay mesh repairs, six (13%) retrorectus mesh repairs, three (6%) suture repairs, one (2%) change of stoma location and one (2%) repair with an intra-abdominal funnel-shaped mesh (Dynamesh IPST™, FEG Textiltechnik, Aachen, Germany), which were all excluded from the analysis due to heterogeneity and the small number of each method used. The contribution of each hospital is stated in Table 1.

**Table 1:** Hospital contribution and technique used

	All	Intra-abdominal keyhole	Sugarbaker
Hospital 1	13(46)	12	1
Hospital 2	9 (32)	4	5
Hospital 3	2 (7)	0	2
Hospital 4	2 (7)	1	1
Hospital 5	2 (4)	1	1
All	28 (100)	18 (64)	10 (36)

Nominal variables are reported as counts and percentages (in parentheses). The percentage indicates the operated portion of each technique

The median follow-up time after primary parastomal hernia repair was 30 (21–64) months. The median follow-up time after Sugarbaker repair was 24 (13–36) months compared with 41 (25–67) months after keyhole repair (Fig. 1). Data of over 24 months follow-up was available for 19 patients, of which 14 were operated on by keyhole and 5 by the Sugarbaker technique. The patients were operated on by 14 surgeons, including gastrointestinal and general surgeons, as well as urologists. Urologists operated on 29% (8/28) of all PSH repairs, while gastrointestinal surgeons operated on 64% (18/28) and general surgeons on 7% (2/28). Concomitant incisional hernias were detected in 18% (5/28) of operations. The PSH repair was accomplished by laparoscopy in 68% (19/28) of operations. The patient characteristics and operations are summarized in Table 2.

**Table 2:** Baseline characteristics and operation

	N total (n = 28)	Intra-abdominal keyhole (n = 18)	Sugarbaker (n = 10)
Age (years)	28	70 ± 9	77 ± 6
Sex	28		
Females		10 (56)	4 (40)
Males		8 (44)	6 (60)
Body mass index (kg/m^2^)	22	28 ± 5	28 ± 4
Follow-up (months)	28	49 ± 34	27 ± 21
Time (months) from primary operation to hernia repair	28	65 ± 33	66 ± 44
Time (months) from hernia repair to recurrence	5	29 ± 12	15
Technique	28		
Laparoscopic		11 (61)	8 (80)
Open		7 (39)	2 (20)
Operation duration (min)	19	94 ± 28	141 ± 46
Blood loss (ml)	26	54 ± 72	39 ± 40
Size of the mesh (cm^2^)?	24	268 ± 80	247 ± 148
Co-existing ventral hernia	28	2 (11)	3 (30)
Fascial closure at the operation	28	12 (67)	7 (70)
Length of stay in the hospital (day)	28	17.8 ± 50.1	6.3 ± 3.7
Operator	28		
Gastrointestinal surgeon		13 (72)	5 (50)
Urologist		4 (22)	4 (40)
General surgeon		1 (6)	1 (10)

Nominal variables are reported as counts and percentages (in parentheses); continuous variables are reported as the mean and standard deviation

The overall PSH recurrence rate during the follow-up was 18% (5/28). The recurrence rates were 22% (4/18) and 10% (1/10) intra-abdominal keyhole and Sugarbaker techniques, respectively. The mean time from primary repair to diagnosed recurrence was 22 months (range 7–42, SD 13 months). Twenty-four months after primary repair, three out of five recurrencies had been diagnosed. Reoperations after repair of a parastomal hernia were undertaken in 5 of these 28 patients during the follow up time. All patients who underwent reoperation were primarily treated by the keyhole technique. Recurrence was the indication for four reoperations. One re-operation was indicated by small bowel perforation as a Clavien–Dindo 4 complication of primary parastomal hernia repair and one by intra-abdominal bleeding as Clavien–Dindo 3b complication. Infectious complication during occurred in 15% (4/28) of patients, with some patients encountering more than one complication; three patiens had postoperative Clavien–Dindo 2 pneumonia and one patient had a Clavien–Dindo 2 urinary infection (Table 3).

**Table 3:** Parastomal hernia repair results

	Intra-abdominal keyhole (n = 18)	Sugarbaker (n = 10)	
Recurrence	4 (22)	1 (10)	
Reoperation	5 (28)	0	
Parastomal hernia recurrence	4 (22)	0	
Complications			
Clavien–Dindo 4 small bowel perforation	1 (6)	0	
Clavien–Dindo 3b bleeding	1 (6)	0	
Clavien–Dindo 2 surgical site infection	0	1 (10)	
Clavien–Dindo 2 urinary infection	1 (6)	0	
Clavien–Dindo 2 pneumonia	3 (17)	0	

Nominal variables are reported as counts and percentages (in parentheses)

## Discussion

Although PSH is a frequent complication after cystectomy [2], hardly any knowledge exists of its repair using intra-abdominal keyhole and Sugarbaker techniques [7, 8]. Moreover, ileal conduit PSHs are still repaired using heterogenous techniques and mesh locations. Therefore, more evidence-based treatment options are demanded for the optimal care of these patients.

The traditional intra-abdominal keyhole technique may not be optimal for PSH repair at the ileal conduit due to its high recurrence rate (22%), as seen in this cohort. The rate is comparable to that published in the meta-analysis [2]. However, the previous literature on the results of intra-abdominal keyhole repair is very limited [2, 7]. Sugarbaker repair has been considered unsuitable for PSH repair following ileal conduit urinary diversion due to posterior attachment of the conduit by ureters, leading to possible difficulties in mobilization [2]. Patients in this cohort were successfully operated on with a low recurrence rate of 10% without increased incidence of complications compared with the keyhole technique. Previous literature on Sugarbaker repair following urinary diversion to compare to is very limited [2, 8].

Elective PSH repair at the ileal conduit is a rarely performed procedure. A nationwide cohort with all major hospitals in Finland concluded with only 28 patients operated on in 2007–2017 by either keyhole or Sugarbaker techniques. Centralization has been proven to improve the quality of care [10]. Despite that, 28 repairs in this cohort were operated on by 14 surgeons.

Perhaps due to the rarity of the repair, the PSH operation was complicated for 15% of patients, some of who encountered more than one complication (Table 3). Additionally, 18% of patients were operated on during the follow-up period, mainly due to recurrence. More research is demanded on intra-abdominal techniques to improve the results. All efforts should be focused on primary repair to gain an acceptable rate of recurrence, complication and re-operation. Additionally, as PSH is a frequent complication of ileal conduit and the results of PSH repair are poor, future studies are encouraged to discover the advantages of PSH prevention [11–13].

This study is limited regarding its retrospective non-randomized manner, small study population and short follow-up time after a Sugarbaker repair. Therefore, no statistical analyses were made. Thus, the results presented here should be considered preliminary and warrant further study. The strength of the study is the nationwide multicenter data inclusion leading to the reliable representation of results in PSH repair following ileal conduit urinary diversion in Finland.

## Conclusions

The results of ileal conduit PSH repair are far below optimal, with high rates of recurrence, re-operations and complications. The Sugarbaker technique may result in fewer recurrences compared with the keyhole technique. Additionally, the Sugarbaker technique seems to be also feasible with ileal conduit parastomal hernia repairs. However, these hypotheses require further research to be conclusive. International studies and registries are required to compare the different methods in PSH repair following ileal conduit urinary diversion due to its rarity.

## Data Availability

The datasets generated and/or analyzed during the current study are not publicly available due to Finnish laws on privacy protection but are available from the corresponding author on reasonable request.

## Abbreviations

SD: Standard deviation
PSH: Parastomal hernia
ICD-10: International Statistical Classification of Diseases and Related Health Problems
BMI: Body mass index
